# Supplementary material for: Microengineered human blood–brain barrier platform for understanding nanoparticle transport mechanisms
Source: Nat Commun. 2020 Jan 10;11:175. doi: 10.1038/s41467-019-13896-7 (PMC6954233; doi:10.1038/s41467-019-13896-7)
Supplement: Supplementary file 1 — Supplementary Information [file 41467_2019_13896_MOESM1_ESM.pdf]

Supplementary Information

Microengineered human blood-brain barrier platform for understanding nanoparticle transport mechanisms

Ahn *et al*.

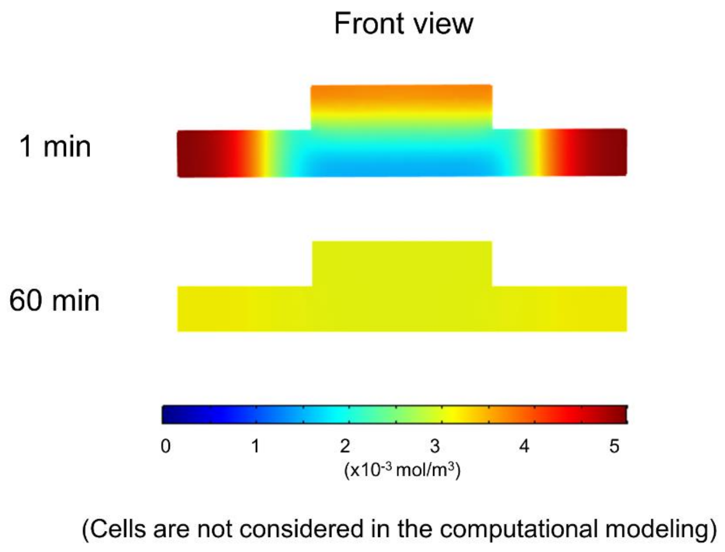

Supplementary Figure 1. Computational fluid dynamic simulation of diffusion through the microfluidic channels in the device. This model has not considered the effect of cells cultured in the device but has simply focused on diffusive transport into the hydrogel channel (lower center channel) in this hybrid channel configuration.

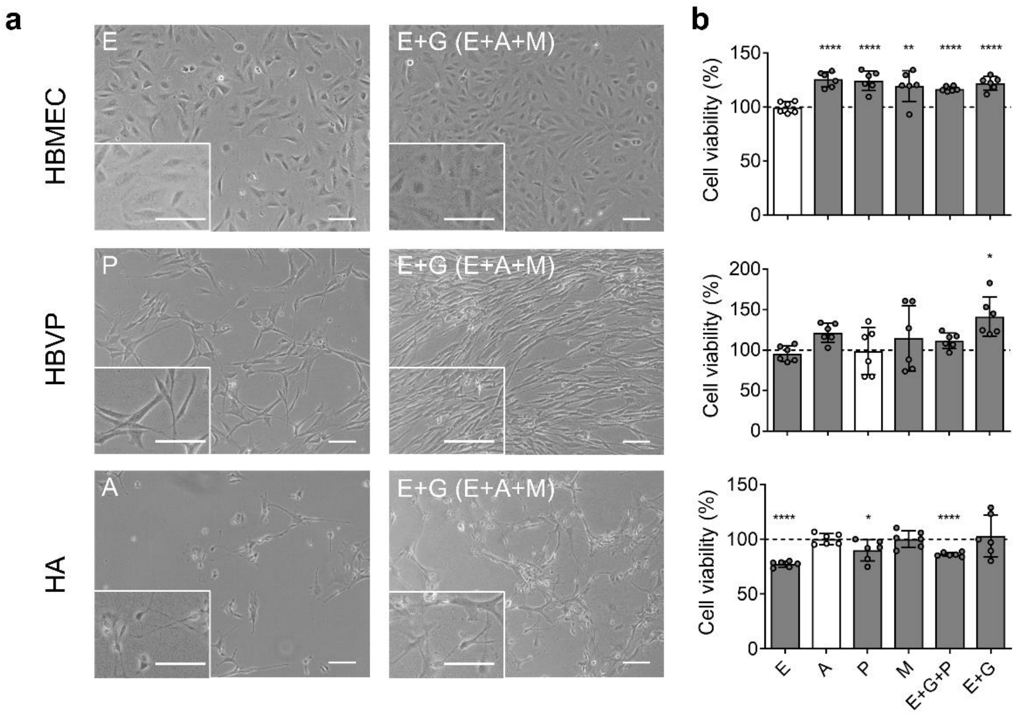

Supplementary Figure 2. Metabolic activities of each cell in different culture medium for co-culture medium selection. a, Morphologies of HBMEC, HBVP, and HA in their respective culture medium and mixed medium (E+G). **b,** Metabolic activities of HBMEC, HBVP, and HA cultured in Endothelial cell medium (E), Astrocyte medium (A), Pericyte medium (P), Microglia medium (M), E+ G (E:A:M= 1:1:1:1), and E+P+G (E:P:A:M=1:1:1:1) (*p<0.05, **p<0.01, and ****p<0.001). Data represent mean ± s.d.

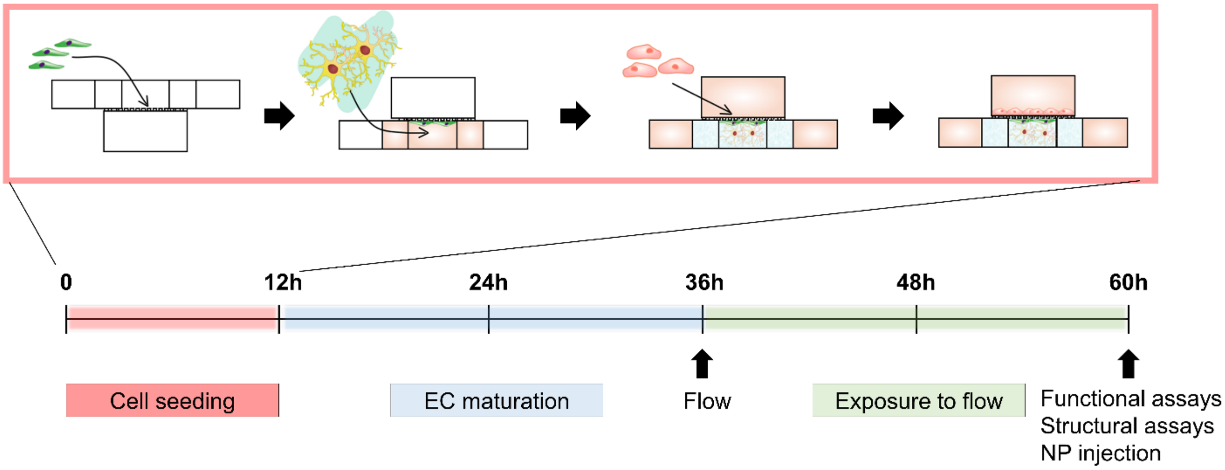

Supplementary Figure 3. Timeline of the experiments. The procedure for cell seeding into the device is as follows: 1. Culture HBVPs on a porous membrane in the abluminal region, while the device is flipped. 2. Inject a glial cells-embedded hydrogel in the abluminal center channel. 3. After 12 hours, culture HBMECs on a porous membrane in the luminal side for additional 1 day. 4. Apply shear flow into the luminal channel for 1 day to establish a tightly connected monolayer.

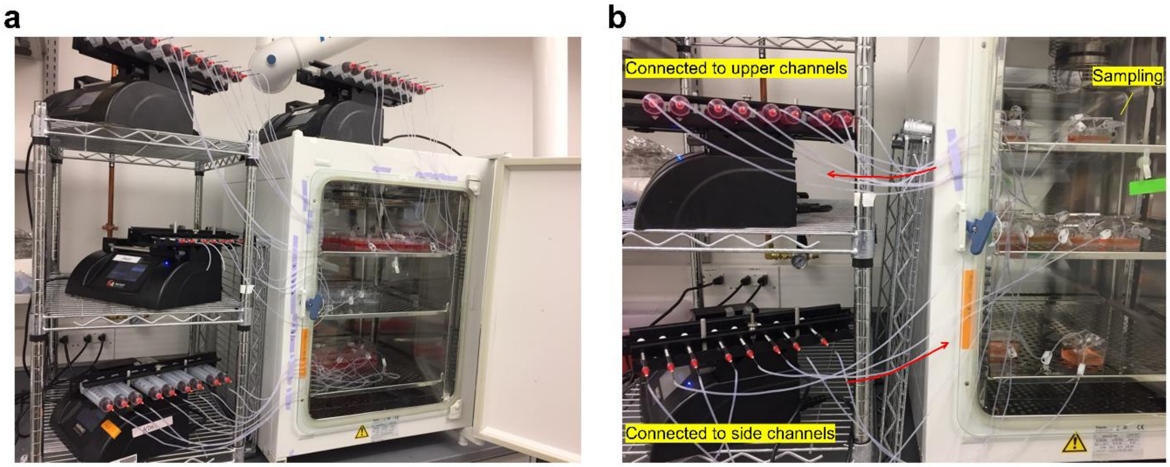

Supplementary Figure 4. Experimental setup. a, Thirty chips in one experimental setup using multi syringe racks. **b,** Experimental setup for permeability assay and molecular sampling.

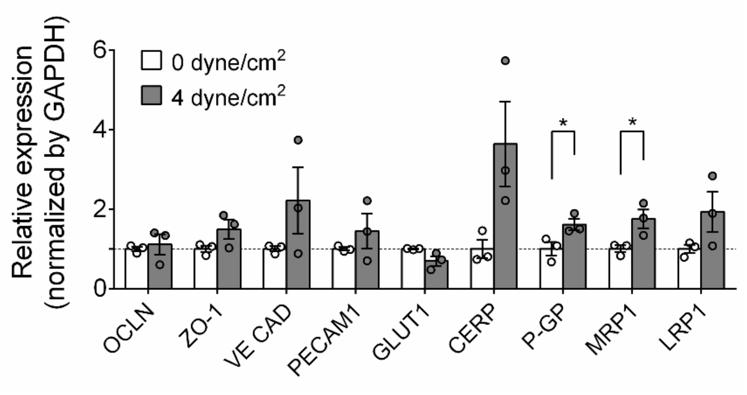

Supplementary Figure 5. Gene expressions of HBMECs in monoculture under static condition (Transwell) and physiological level of shear stress (chip, 4 dyne/cm^2). Data represent mean ± s.e.m.

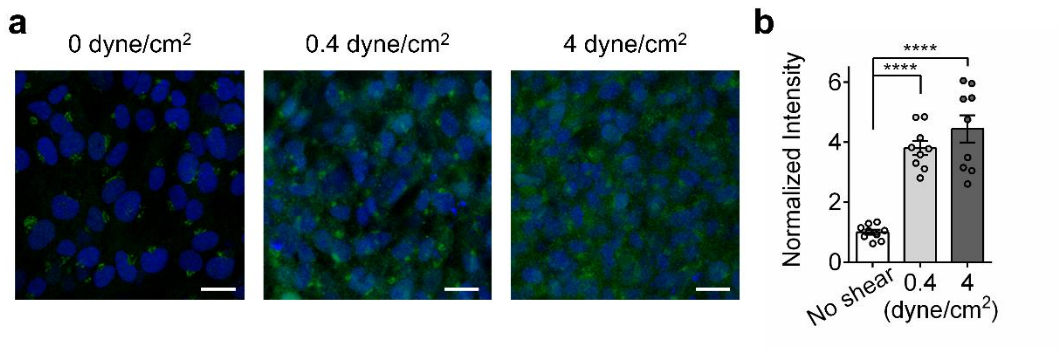

Supplementary Figure 6. Endothelial nitric oxide synthase (eNOS) phosphorylation in HBMECs under different levels of shear stress. a, Confocal images of HBMECs from different shear stress conditions labelled with phospho-eNOS (Ser1177) (eNOS, green; DAPI, blue) (scale bars = 20 µm). **b**, Fluorescence intensities of eNOS from confocal images normalized by the average intensity of the static condition (n=9 for each condition, ****p<0.001). Data represent mean ± s.e.m.

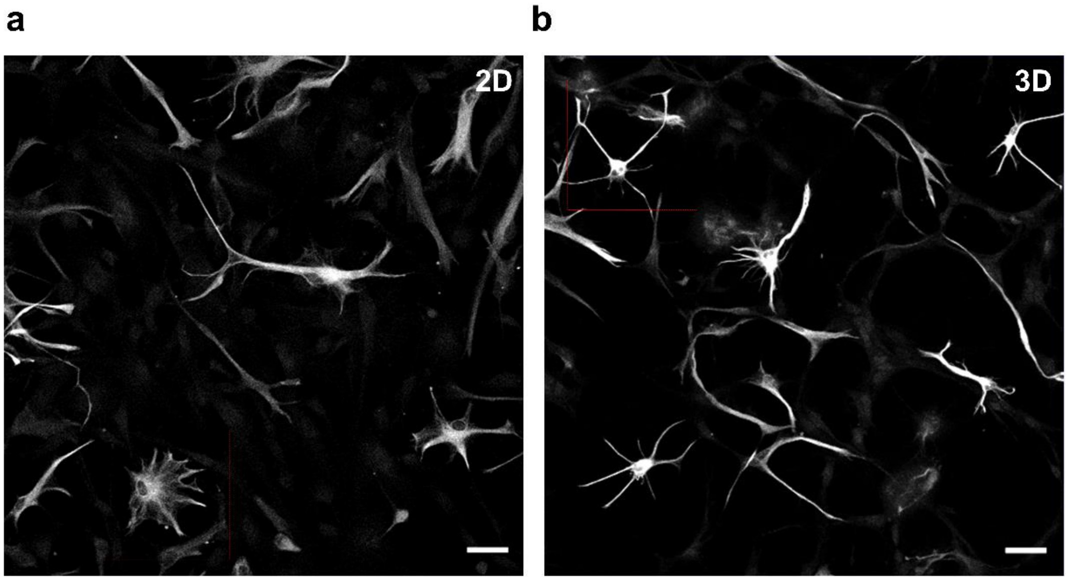

Supplementary Figure 7. Morphologies of HAs cultured on 2D Matrigel coated surface and in 3D Matrigel. a, HAs cultured on 2D Matrigel coated surface showed flat polygonal shapes. **b,** HAs cultured in 3D Matrigel exhibited small cell bodies with radial distribution of long cellular processes. Scale bars = 50 µm.

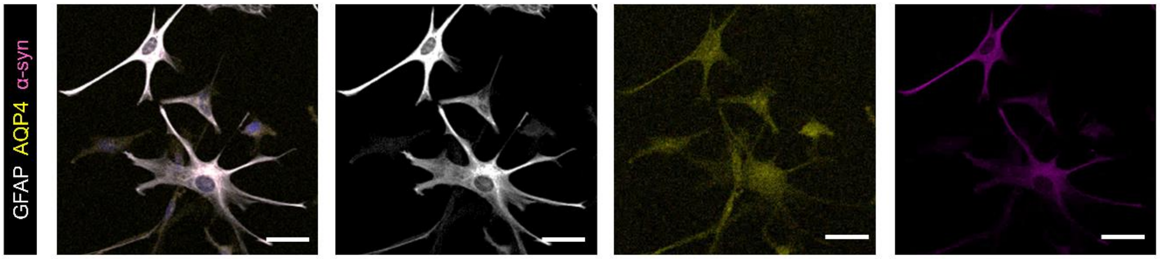

Supplementary Figure 8. Aquaporin-4 (AQP4) and α-syntrophin (α-syn) expression in 2D cultured astrocytes. Confocal images of 2D monoculture of astrocytes (GFAP, white) showing diffusive expression of AQP4 (AQP4, yellow) and α-syn (α-syn, magenta). Scale bars = 50 µm.

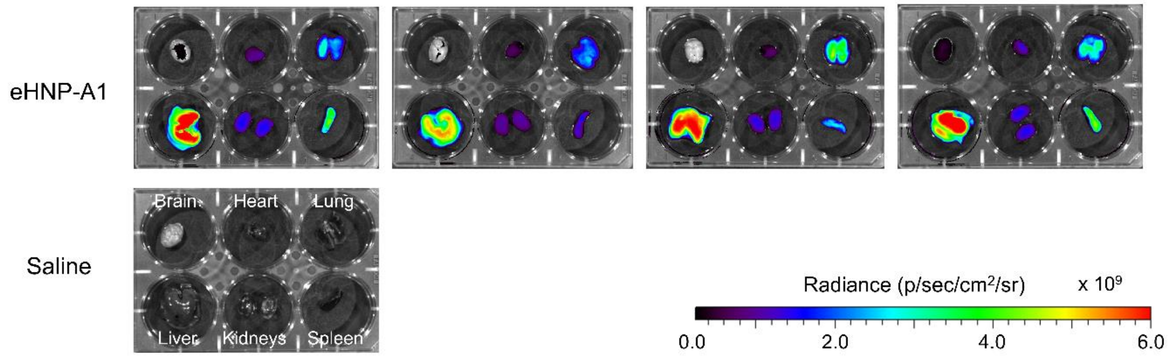

Supplementary Figure 9. *Ex-vivo* biodistribution of eHNP-A1. Organ distribution of dye-loaded eHNP-A1 24 h after intravenous administration. Fluorescence signals of eHNP-A1 were detected in the brains as compared to the saline control.

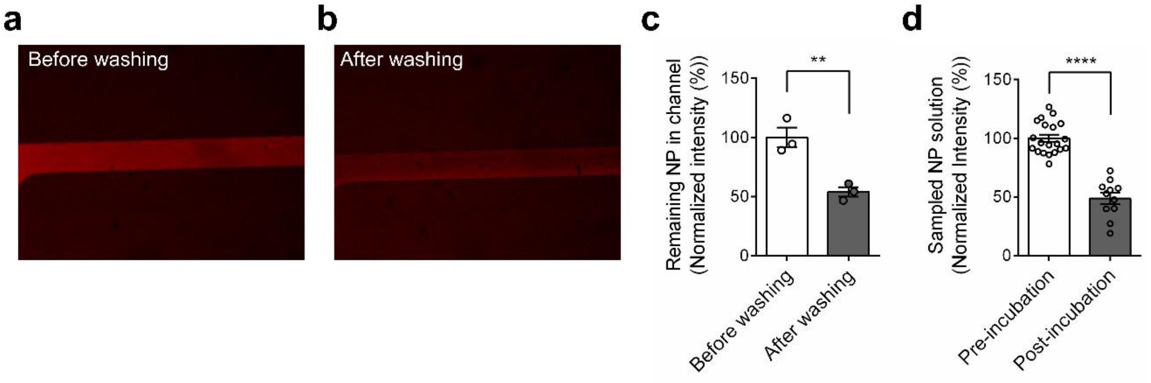

Supplementary Figure 10. eHNP-A1 loss in a microfluidic channel due to the adsorption to the PDMS surface. a,b, Fluorescent images showing eHNP-A1 solution inside the vascular channel after 2 h of NP incubation before washing the channel (**a**) and after washing the channel with PBS (**b**). Single layer of the vascular channel without cells were used to measure the eHNP-A1 loss caused by adsorption to the PDMS. **c,** Fluorescence intensities in the vascular channel in images quantified using ImageJ, indicating the remaining NPs in the channel. The fluorescence intensities were normalized to that from the channel before washing (n=3 for each condition, $^{**}p<0.01$). **d,** Fluorescence intensities of NP solutions before injecting into the microchannel (pre-incubation) and sampled from the microchannel after 2 h of incubation (post-incubation) measured with a plate reader, indicating working concentration of the NP solution (n=20 for pre-incubation and n=11 for post-incubation, $^{****}p<0.001$). The intensities were normalized to those of the pre-incubated NP solution. Data represent mean ± s.e.m.

Experiment	Gene Symbol	Gene Name	TaqMan Assay ID #
	GAPDH	Glyceraldehyde-3-phosphate dehydrogenase	Hs02786624_g1
HA RT-qPCR	GFAP	Glial fibrillary acidic protein	Hs00909233_m1
	VIM	Vimentin	Hs00958111_m1
	LCN2	Lipocalin-2	Hs01008571_m1
HBMEC Fluidigm	vWF	von Willebrand factor	Hs01109446_m1
	SELE	Selectin E	Hs00174057_m1
	PECAM1	Platelet and endothelial cell adhesion molecule 1	Hs01065279_m1
	VECAD	Cadherin 5 (CDH5)	Hs00901465_m1
	OCLN	Occludin	Hs00170162_m1
	ZO-1	Tight junction protein 1 (TJP1)	Hs01551861_m1
	CAT1	Solute carrier family 7 member 1 (SLC7A1)	Hs00931450_m1
	LAT1	Solute carrier family 7 member 5 (SLC7A5)	Hs00185826_m1
	OCT1	Solute carrier family 22 member 1 (SLC22A1)	Hs00427552_m1
	GLUT1	Solute carrier family 2 member 1 (SLC2A1)	Hs00892681_m1
	CERP	ATP binding cassette subfamily A member 1 (ABCA1)	Hs01059137_m1
	P-GP	ATP binding cassette subfamily B member 1 (ABCB1)	Hs00184500_m1
	MRP1	ATP binding cassette subfamily C member 1 (ABCC1)	Hs01561483_m1
	LRP1	LDL receptor related protein 1	Hs00233856_m1
	AGER	Advanced glycosylation end-product receptor	Hs00542584_g1
	ICAM1	Intercellular adhesion molecule 1	Hs00164932_m1
	VCAM1	Vascular cell adhesion molecule 1	Hs01003372_m1

Supplementary Table 1. Primers/probes information. The target genes were assessed using commercially available primers and probes.

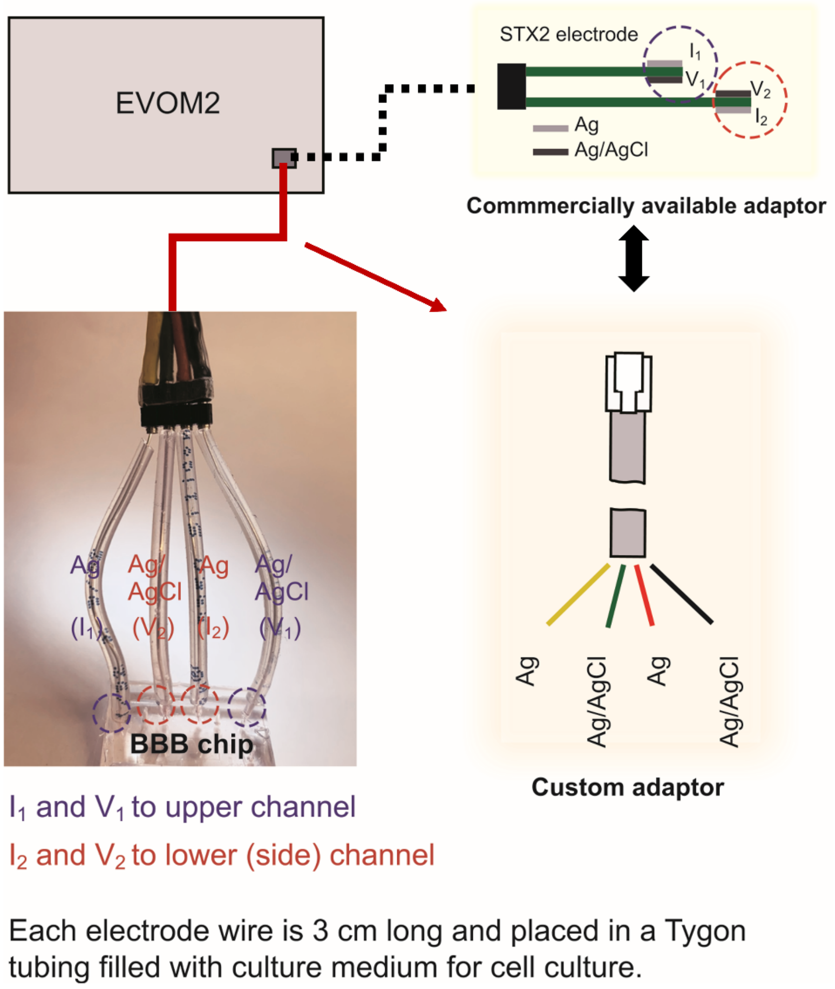

Supplementary Figure 11. Transendothelial electrical resistance (TEER) measurement. TEER was measured using a commercially available volt-ohmmeter (EVOM2) with a custom electrode adaptor made with Rj11 plug and Ag, Ag/AgCl electrode wires. The 3 cm electrode wires were placed in a tygon tubing filled with culture medium to reduce the possible background resistance and error as previously reported.[1] Our custom electrode adaptor has four ports connected to the four Rj11 leads (two ports are connected to Ag wires to pass current and the other two ports are connected to Ag/AgCl wires to detect voltage). Each of the Ag and Ag/AgCl wire is inserted into the upper (indicated by blue circles) and the lower side channel (indicated by red circles).

#	EC only	BBB (4 dyne/cm^2)	BBB (No shear)	BBB (0.4 dyne/cm^2)
**1**	7320	10373	7485	8960
**2**	7127	9712	6309	6974
**3**	9407	10399	7030	9175
**4**	9515	9782	7703	9372
**5**	5690	9412	6767	
**6**	7907	9217		
**7**	9059	9441		
**8**	9522	9476		
**9**	9659	9734		
**10**	5835	9107		
**11**	9583	9311		
**12**		9528		

unit: Ω

Supplementary Table 2. Raw resistance data measured using EVOM2. Raw resistance data of the endothelial monolayer only model (EC only) and the BBB model (BBB) (under different shear stress levels). The data may not be linearly correlated to the actual cell layer resistance values due to the inhomogeneous potential distribution over the channel length.

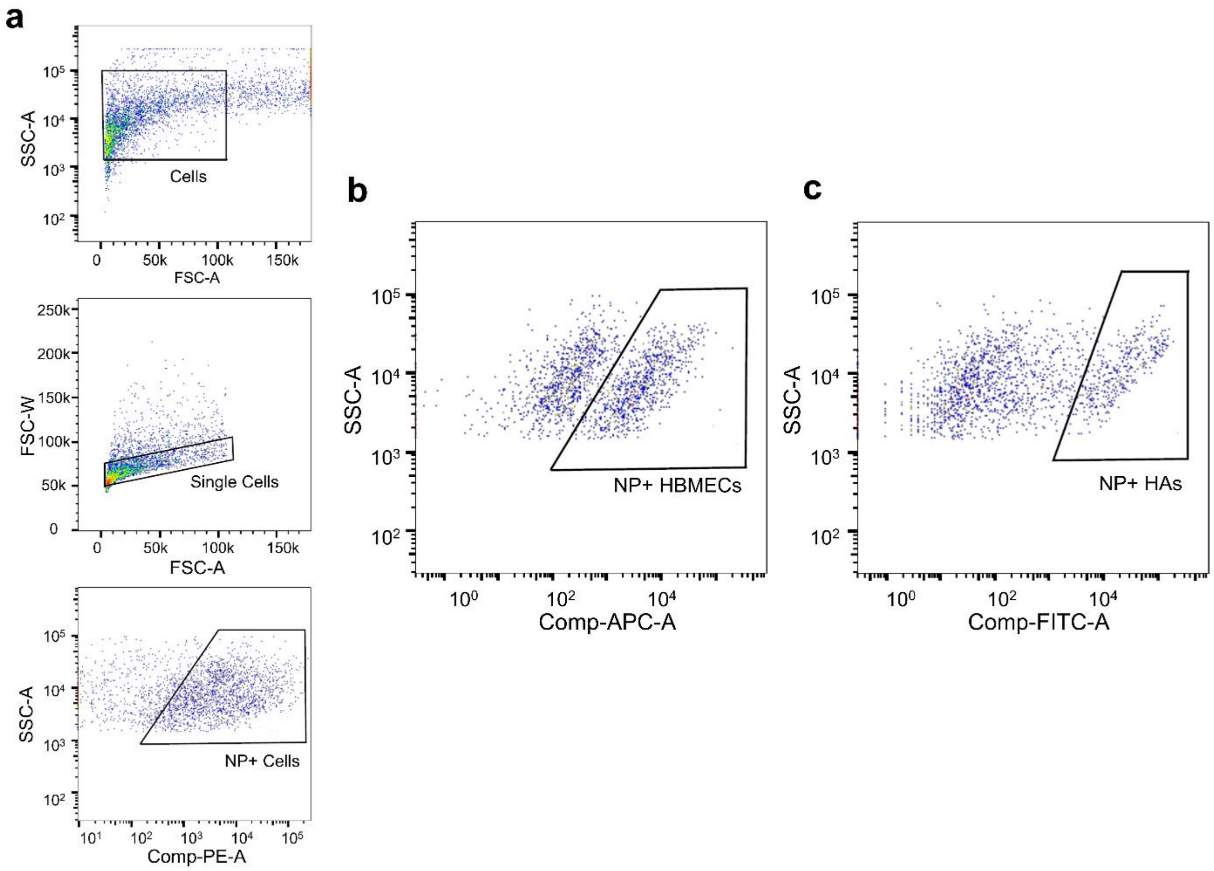

Supplementary Figure 12. Example of gating strategy for flow cytometric analysis of eHNP-A1^{+} cells. a, Cell suspensions were hierarchically gated as follows: Cells were gated and debris were excluded using FSC-A/SSC-A. Single cells were selected using FSC-A/FSC-W gate. NP^{+} cells were distinguished with PE fluorescence. **b,** PE^{+}/APC^{+} cells were considered as NP^{+} HBMECs. **c,** PE^{+}/FITC^{+} cells were considered as NP^{+} HAs.

References

1 Kim, Y. *et al.* Probing nanoparticle translocation across the permeable endothelium in experimental atherosclerosis. *Proc Natl Acad Sci U S A* **111**, 1078-1083 (2014).
