# Supplementary material for: Microengineered human blood–brain barrier platform for understanding nanoparticle transport mechanisms
Source: Nat Commun. 2020 Jan 10;11:175. doi: 10.1038/s41467-019-13896-7 (PMC6954233; doi:10.1038/s41467-019-13896-7)
Supplement: Supplementary file 3 — Reporting Summary [file 41467_2019_13896_MOESM3_ESM.pdf]

nature research

Corresponding author(s): NCOMMS-19-12073C

Last updated by author(s): Nov 25, 2019

Reporting Summary

Nature Research wishes to improve the reproducibility of the work that we publish. This form provides structure for consistency and transparency in reporting. For further information on Nature Research policies, see Authors & Referees and the Editorial Policy Checklist .

Statistics

For all statistical analyses, confirm that the following items are present in the figure legend, table legend, main text, or Methods section.

n/a	Confirmed	
☐	☒	The exact sample size (*n*) for each experimental group/condition, given as a discrete number and unit of measurement
☐	☒	A statement on whether measurements were taken from distinct samples or whether the same sample was measured repeatedly
☐	☒	The statistical test(s) used AND whether they are one- or two-sided<br>*Only common tests should be described solely by name; describe more complex techniques in the Methods section.*
☐	☒	A description of all covariates tested
☐	☒	A description of any assumptions or corrections, such as tests of normality and adjustment for multiple comparisons
☐	☒	A full description of the statistical parameters including central tendency (e.g. means) or other basic estimates (e.g. regression coefficient) AND variation (e.g. standard deviation) or associated estimates of uncertainty (e.g. confidence intervals)
☐	☒	For null hypothesis testing, the test statistic (e.g. *F*, *t*, *r*) with confidence intervals, effect sizes, degrees of freedom and *P* value noted<br>*Give P values as exact values whenever suitable.*
☒	☐	For Bayesian analysis, information on the choice of priors and Markov chain Monte Carlo settings
☒	☐	For hierarchical and complex designs, identification of the appropriate level for tests and full reporting of outcomes
☒	☐	Estimates of effect sizes (e.g. Cohen's *d*, Pearson's *r*), indicating how they were calculated

Our web collection on statistics for biologists contains articles on many of the points above.

Software and code

Policy information about availability of computer code

Data collection	Comfocal images were collected with Zen2 blue edition (2.0.14283.202, Zeiss).<br>Real-time quantitative PCR data were collected with StepOne (2.0, Applied Biosystems) and Biomark HD (4.5.1, Fluidigm).<br>Fluorescent data from mice organs were collected with Living Image (4.4.5, PerkinElmer).<br>Fluorescent data from solutions were collected with Gen5 (2.07, BioTek).<br>Flow-cytometry data were collected with BD FACS Aria III (BD Biosciences).
Data analysis	Graphpad Prism 6 was used to generate plots for quantification data and statistical analysis (t-tests).<br>Comsol (5.3a, COMSOL) was used to model serum transport in a microfluidic device.<br>Zen2 blue edition (2.0.14283.202, Zeiss) was used to analyze confocal images.<br>ImageJ (1.50i, NIH) was used to calculate fluorescence intensity, measure cell body sizes, and measure cell process lengths.<br>Living Image (4.4.5, PerkinElmer) was used to quantify fluorescent data from mice organs.<br>FlowJo (V10, FlowJo LLC) was used to generate FACS plots.

For manuscripts utilizing custom algorithms or software that are central to the research but not yet described in published literature, software must be made available to editors/reviewers. We strongly encourage code deposition in a community repository (e.g. GitHub). See the Nature Research guidelines for submitting code & software for further information.

Data

Policy information about availability of data

All manuscripts must include a data availability statement. This statement should provide the following information, where applicable:

- Accession codes, unique identifiers, or web links for publicly available datasets
- A list of figures that have associated raw data
- A description of any restrictions on data availability

The authors declare that all data supporting the results in this study are available within the paper and its Supplementary Information. All raw-data is available upon request.

Field-specific reporting

Please select the one below that is the best fit for your research. If you are not sure, read the appropriate sections before making your selection.

[x] Life sciences   [ ] Behavioural & social sciences   [ ] Ecological, evolutionary & environmental sciences

For a reference copy of the document with all sections, see nature.com/documents/nr-reporting-summary-flat.pdf

Life sciences study design

All studies must disclose on these points even when the disclosure is negative.

Sample size	The sample sizes were equal or larger than 3 in all cases, which are similar to those generally employed in the field. No method was used to predetermine the sample sizes.
Data exclusions	Data was not excluded from experiments unless apparent failures, such as cell death due to contamination during the experimental period.
Replication	All experiments were replicated with at least 3 chip set-ups, and all attempts at replication were successful.
Randomization	Samples were allocated into experimental group at random.
Blinding	The investigators were blinded to group allocation during experiments and data collection.

Reporting for specific materials, systems and methods

We require information from authors about some types of materials, experimental systems and methods used in many studies. Here, indicate whether each material, system or method listed is relevant to your study. If you are not sure if a list item applies to your research, read the appropriate section before selecting a response.

Materials & experimental systems

n/a	Involved in the study	
[ ]	[x]	Antibodies
[ ]	[x]	Eukaryotic cell lines
[x]	[ ]	Palaeontology
[ ]	[x]	Animals and other organisms
[x]	[ ]	Human research participants
[x]	[ ]	Clinical data

Methods

n/a	Involved in the study	
[x]	[ ]	ChIP-seq
[ ]	[x]	Flow cytometry
[x]	[ ]	MRI-based neuroimaging

Antibodies

Antibodies used	Immunochemical staining<br>Goat anti-ZO1 (1:200; Abcam)<br>Mouse anti-GFAP (1:200; Invitrogen)<br>AlexaFluor 488 conjugated Rabbit anti-a-SMA (1:200; Abcam)<br>Rabbit anti-AQP4 (1:200; Invitrogen)<br>Rabbit anti-a-SMA (1:200; Abcam)<br>Donkey anti-Goat AlexaFluor 633 (1:200; Invitrogen)<br>Chicken anti-Mouse AlexaFluor 594 (1:200; Invitrogen)
Validation	Only company-validated antibodies were used in this work for immunocytochemistry and immunohistochemistry analyses.

Eukaryotic cell lines

Policy information about cell lines

Cell line source(s)	Human brain microvascular endothelial cell line was purchased from Innoprot (P10361-IM).
Authentication	Only company-validated cell line was used in this work.
Mycoplasma contamination	The cell line (immortalized human brain microvascular endothelial cells) was tested negative for mycoplasma contamination, which was performed by company.
Commonly misidentified lines (See ICLAC register)	No commonly misidentified cell lines were used in this study.

Animals and other organisms

Policy information about studies involving animals; ARRIVE guidelines recommended for reporting animal research

Laboratory animals	4-5 week of male balb/c mice (Jackson Labs, city, state, USA) were given an irradiated dietary regiment until the mice were 20-21 weeks of age for experiment.
Wild animals	The study did not involve wild animals.
Field-collected samples	The study did not involve samples collected from the field.
Ethics oversight	All procedures were performed in accordance with the approval of the Institutional Animal Care and Use Committee (IACUC) at Georgia Institute of Technology.

Note that full information on the approval of the study protocol must also be provided in the manuscript.

Flow Cytometry

Plots

Confirm that:

- [x] The axis labels state the marker and fluorochrome used (e.g. CD4-FITC).
- [x] The axis scales are clearly visible. Include numbers along axes only for bottom left plot of group (a 'group' is an analysis of identical markers).
- [x] All plots are contour plots with outliers or pseudocolor plots.
- [x] A numerical value for number of cells or percentage (with statistics) is provided.

Methodology

Sample preparation	Fluorescently labeled cells were isolated from microfluidic devices, fixed with 4% PFA, and collected in ice-cold FACS buffer (PBS with 2% FBS).
Instrument	Data for the flow-cytometry experiments were collected using BD FACS ARIA III
Software	BD FACSDiva was used to collect individual cell information during sorting and FlowJo was used to analyze data and produce plot.
Cell population abundance	No sorting experiments were done in these studies. For flow-cytometry analysis, each sample contained 10^4 viable cells. After staining, 5x10^3 total events were recorded for each sample before statistical tests were performed.
Gating strategy	Single cells were collected by drawing FSC/SSC gates. APC gate was used to detect HBMECs, FITC gate was used to detect HAs, and PE gate was used to obtain NP positive cells.

- [x] Tick this box to confirm that a figure exemplifying the gating strategy is provided in the Supplementary Information.
